# Evaluation of Ameliorative Potential of Vitamins E and C on DNA Double Strand Break (DSB) in Patients Undergoing Computed Tomography (CT): A Clinical Study

**DOI:** 10.22088/IJMCM.BUMS.7.4.226

**Published:** 2019-03-04

**Authors:** Seyed Masoud Jafarpour, Morteza Salimian, Mehran Mohseni, Hamid Reza Talari, Akbar Aliasgharzadeh, Bagher Farhood, Habiballah Moradi, Hossein Safari

**Affiliations:** 1 *Department of Radiology and Medical Physics, Faculty of Paramedicine, Kashan University of Medical Sciences, Kashan, Iran.*; 2 *Department of Medical laboratory, Faculty of Paramedicine, Kashan University of Medical Sciences, Kashan, Iran.*; 3 *Department of Radiology, Kashan University of Medical Sciences, Kashan, Iran.*; 4 *Health Promotion Research Center, Iran University of Medical Sciences, Tehran, Iran.*

**Keywords:** Computed tomography (CT), DNA double strand break (DSB), Vitamin C, Vitamin E

## Abstract

Computed tomography (CT) is one of the most important diagnostic X-ray procedures which plays an important role in increasing the patient dose values. The purpose of this clinical study was to evaluate the efficacy of vitamins E and C in lowering down the level of DNA double strand break (DSB) caused by CT scan. Sixty patients for abdomen/pelvic enhanced CT scan were randomly assigned to placebo (control), vitamin C, and vitamin E groups. The patient blood samples were taken before and immediately after the CT scan. Counting the number of DSB was performed using γ-H2AX method as a sensitive biomarker. Immediately after the CT scan, the mean number of DSBs/cell increased in all three groups of control (131%, P<0.001), vitamin C (103%, P <0.001), and vitamin E (66%, P<0.001) compared to their mean before the CT scan. Furthermore, the results showed that vitamin E decreased the mean number of DSBs/cell by 22% in comparison with the control group (P =0.023), whereas vitamin C had no significant effect on reducing the DSB (<3%, P =0.741). It is concluded that the administration of vitamin E one hour before the CT scan, significantly decreases DSB levels.

After invention of the first computed tomography (CT) scanner in 1963, its application increased significantly among X-ray diagnostic examinations, mainly due to its capabilities (1). Currently, a large fragment of diagnostic X-ray dose to the patients results from CT scan (2-4). It is estimated that nearly 85 million CT scans are performed each year in the United States which is almost 20 times higher in comparison to the previous decades (5-7). The annual increase rate of CT scan usage is approximately 10% yearly, and it currently accounts for over 50% of the population’s radiation exposure (8). Endogenous oxidative stress after radiation exposure induces different DNA damages including base damage, single- strand breaks (SSB), and double- strand breaks (DSB). Any defect in the repair of DSB can lead to genetic damage resulting in malignancy in the future (9,10). Phosphorylation of histone variant (H2AX) is a very sensitive biomarker for the quantification of DSBs in peripheral blood lymphocytes. Immunofluorescence microscopy of this phosphorylated histone, known as γ-H2AX, can be used to screen the DSB induction and reparation (11). CT data have shown that the number of γ-H2AX depends linearly on the dose-length product (DLP) (10-12); hence, increasing the patient’s dose would increase the number of DSBs.

In addition, many imaging modalities, such as angiography, urography or CT need to use iodinated contrast medium before and during the irradiation, while allergic reactions and kidney failure of these contrast media have been reported in previous studies (13,14). Furthermore, it has been reported that administration of iodinated contrast medium increases the DSB in peripheral blood lymphocytes (15-18).

Moreover, antioxidants have been proven to be effective in reducing the harmful effects of radiation (19-21). It has been shown that different antioxidants reduce the number of DSBs in diagnostic dose range (22-24). However, there are few studies which have assessed the effects of antioxidants on radiation damages induced by diagnostic dose and their abilities have only been proven in in vitro studies (18, 22, 25, 26). Hence, the current study aimed to investigate the effect of vitamins C and E on the number of γ-H2AX foci in blood lymphocytes of patients undergoing diagnostic CT examinations with iodinated contrast medium.

## Materials and Methods

**Study design**

The current study was carried out after obtaining the approval from the ethics review board of Kashan University of Medical Sciences (IR.KAUMS.REC.1394.139) as well as National Registry of Clinical Trial (IRCT2016091029720N2). After describing the implementation procedures of the study by the researchers, all subjects read the participation conditions and gave their written consent. A total of sixty patients (18-65 years old) were selected in Radiology Department of Shahid Beheshti Medical Center. The number of patients enrolled in this study was similar to some other clinical studies on CT procedures (10, 12, 15). All patients were scheduled for abdomen/pelvic enhanced CT, and were classified into three groups of placebo (group A, as control), vitamin C (group B), and vitamin E (group C). The patients with a history of leukemia or lymphoma, blood disorders, former radiation therapy or chemotherapy, a previous history of exposure to any radiological procedures or nuclear medicine studies within 3 days, and smoker patients were excluded from the current study. Demographic data and dosimetric information of the patients participated in this study are listed in Table 1.

The antioxidants used in the current study included vitamins C and E which their concen-trations were selected based on the package insert (PI). Therefore, the dose of vitamins was determined according to the recommended daily intake (RDI) that was also used in a previous study (23); so that it has the minimum recommended intake. Therefore, the final concentrations of vitamins C and E were 500 mg and 400 mg, respectively. For the pre-treatment, 20 patients received vitamin C, 20 patients received vitamin E, and 20 patients received placebo 60 min (± 10 min) before the CT scan.

To obtain DSB base level, the first blood sample from each subject was drawn from the antecubital vein before the CT scan. The blood samples were collected using ethylene diamine tetra acetic acid (EDTA) containing vials. Then, abdomen/pelvic CT scan was performed for all patients using a 16-detector-row CT scanner (Aquilion 16, Toshiba, Japan). During the CT scan, contrast medium was used for all patients and 85 ml (±10 ml) of iodixanol 320 mgI/mL (Visipaque, GE Healthcare) was injected for them. After CT examination, the second blood sample was immediately taken from the patients.

Various clinical parameters were applied according to the patient's physics. Computed tomography dose index (CTDI) measurements were performed using our clinical technique parameters. Then, volume CTDI (CTDIvol) and dose length product (DLP), which represent the total dose received by the patient (27), were calculated for all of the patients.

**Sampling process and immunofluorescence analysis**

**Fig. 1:**
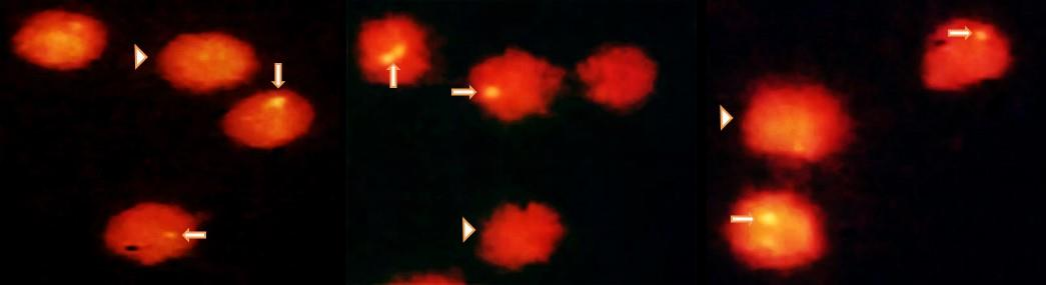
**Visualization of γ-H2AX foci in lymphocytes patients. **Typical γ-H2AX-foci (arrow) and lymphocyte (arrow head) were observed by fluorescence microscopy (x 1000)

The peripheral blood samples (before and after the CT scan) were transported to laboratory at 4 °C and were proceeded quickly. The volume of 2.5 ml of blood was diluted with phosphate buffered saline (PBS) at 1:1 ratio and the lymphocytes were separated by ficollpaque density gradient (Inno- Train, Germany) with centrifugation at 2500 g for 15 min at 4 °C. The cells were washed twice with PBS (each for 5 min) and were fixed with 4% paraformaldehyde (PFA) (Merck, Germany) for 15 min. After washing them two times with PBS, approximately 10 µl of solution was pipetted on slide followed by permeabilization in 100% acetone (Merck, Germany) for 10 min (-20 °C). Then, the cells were washed three times with PBS (each for 5 min) and were blocked in 5% BSA (Sigma, USA) and 0.2% triton X-100 at room temperature. The samples were incubated with 50 μl anti-phosphohistone H2AX monoclonal antibody (clone JBW301, Millipore, Germany) [dilution 1:500 in PBS containing 1% BSA and 0.05% X-triton] for overnight at 4 °C. The slides were washed three times (each for 10 min) and were stained with the secondary antibody Conjugated to Alexa Fluor 488 (Cell Signaling, USA) [dilution 1:500 in PBS containing 1% BSA and 0.05% X-triton] for 1 h at room temperature and in the darkness. Finally, the cells were washed three times with PBS (each for 10 min) and were stained with 10 µl propidium iodide (Invitrogen, USA) at dilution 1:50. A glass coverslip was embedded on top of the samples and was sealed with fingernail polish. In each sample, the numbers of γ-H2AX foci (DSBs) were visually counted over 100 lymphocyte cells using fluorescence microscope (Magnum_T, Ceti Co., UK) and by two blind observers (Figure 1). Granulocytes and monocytes were deleted by morphological criteria and, finally, the average number of foci/cell was calculated.

**Table 1:** Demographic characteristics and dosimetric information of the patients who participated in three groups of control, vitamin C, and vitamin E

Variable	Placebo (control)	Vitamin C	Vitamin E	P-value
Number of patients (n)	20	20	20	-
Female: Male	10:10	8:12	11:9	0.715
Age (mean ± SD)	43±9	41±12	42±12	0.831
Female (mean, range)	36, 18-64	33, 25-43	44, 31-58	-
Male (mean, range)	47, 30-66	46, 21-65	41, 28-65	-
CTDI_vol_ (mean ± SD)	6.68±1.62	5.98±1.48	5.94±1.51	0.734
DLP (mean±SD)	345±88	337±116	326±140	0.880

DLP: dose length product (mGy.cm); CTDI_vol_,: volume computed tomography dose index (mGy); SD: standard deviation.

**Statistical analysis**

This study was performed in completely randomized design experiment. Mean differences of the DSBs/cell values of each group (before and after CT scan) were tested with paired samples t-test and comparisons of change scores between the treated and the control groups were performed by a one-way ANOVA. The significant difference was set at P <0.05. Data were expressed as means ± standard error on the mean (SEM).

## Results

Sixty patients were entered into the study from May 2016 to November 2016. All subjects were randomly divided into three groups. Basic information of the subjects (such as sex, age, and dose value) are shown in Table 1. There was no difference between control and intervention groups in terms of sex, age, and dose value. After ingestion of vitamins, no serious clinical adverse effects were observed in the subjects. The minimum (min) and maximum (max) DLP were 163.9 mGy.cm and 757.8 mGy.cm, respectively, and the mean DLP was recorded as 336.5±114 mGy.cm. 

**Fig. 2:**
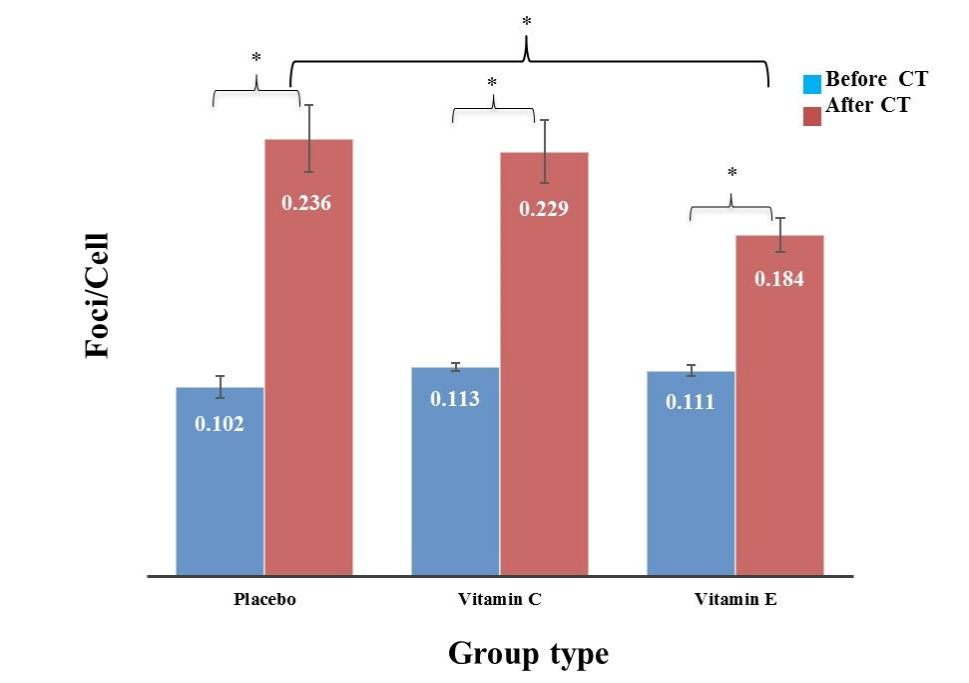
**The average number of γ-H2AX-foci in control, vitamins C and E groups before and after the CT scan.** *represents statistically significant difference. There was a statistically significant difference in number of gamma-H2AX foci/cell before and after the CT scan in control, vitamins C and E groups. Moreover, there was a statistically significant difference in number of gamma-H2AX foci/cell after radiation exposure in the control group compared to the vitamin E group

The findings showed that the background level of DSBs/cell in groups A, B and C were 0.102±0.006, 0.113±0.002, and 0.111±0.003, respectively. There was no significant difference in number of gamma-H2AX foci/cell before radiation exposure in the control group in comparison with the vitamin C group (P = 0.073) and the vitamin E group (P = 0.150) as well as between the vitamin C group and the vitamin E group (P =0.719). After the CT scan, the average number of DSBs/cell increased and the obtained data showed that this increase was significant in all groups (Figure 2). Mean number of DSBs/cell induced by the exposure to CT scan was 0.236±0.018, 0.229±0.017, and 0.184±0.009 in groups A, B, and C, respectively. The recorded number of DSBs/cell for the patients treated with vitamin E (group C) and placebo (control group) showed a significant decrease (P= 0.023), while vitamin C (group B) had no significant effect on the number of DSBs/cell (P= 0.741).

 Moreover, there was a significant difference in number of gamma-H2AX foci/cell before and after the CT scan in control, vitamins C and E groups (P<0.001).

## Discussion

Although recent studies have confirmed that the antioxidant supplementations are effective in reducing the number of DSB, there is little data about the efficacy of these ingredients in diagnostic dose range. Therefore, a randomized, double-blinded, and placebo- controlled trial study was conducted to examine the effect of vitamins C and E on the number of DSBs in blood lymphocytes of patients undergoing diagnostic CT examinations with iodinated contrast medium. In this study, the γ-H2AX method was used to evaluate the radiation-induced genetic damages as it is an easier and more sensitive method compared to the old methods such as comet assay (28). For more than a decade, this technique has been used in diagnostic studies and it is a reliable method for *biological dosimetry* of radiation as it can show the radiation effects of lower dose values (≤1 mGy) (10,11,29-31).

Nowadays, most of the CT examinations are the abdomen/pelvic scans (8). On the other hand, the iodinated contrast medium is applied in most of the diagnostic X-ray examinations (32), which it can increase the number of foci during the radiation due to the photoelectric effect (33-35). Path et al. reported that the number of γ-H2AX increases by 58% immediately after the abdomen/pelvic CT scan in the presence of the iodinated contrast medium (16). Furthermore, increased number of γ-H2AX in the presence of the iodinated contrast medium has been also confirmed in other similar studies (15,17). Hence, in the present study, the abdominal/pelvic CT scan with the iodinated contrast medium was selected as one of the most common diagnostic X-ray examinations.

The results of the current study revealed that the DSB level increases immediately following the CT scan. In a study by Löbrich et al. (10) on the radiation damages induced by CT examinations, it was reported that there is a linear relationship between the number of γ-H2AX foci and DLP value. The number of foci/cell generated in diagnostic examinations reaches the initial level after 24 h, and reparation occurs (10,36); however, any defect in the process of repair can trigger malignancy and carcinogenicity (9).

Using antioxidants can be considered as an effective method in reducing the radiation-induced damages as it scavenges the free radicals produced by the radiation (37,38). The effect of the antioxidants with different concentrations against different radiation dose values has been investigated in several studies (19,20). In a study, the effect of a commercial antioxidant compound was evaluated in two phases of in vitro and in vivo/in vitro for a diagnostic dose of 10 mGy. The final concentrations of this compound in the in vivo/in vitro experiment included 500 mg calcium ascorbate, 400 IU d-alpha tocopheryl succinate, 15 mg natural mixed carotenoids, 250 mg N-acetyl cysteine, 30 mgalpha-lipoic acid, and 100 µg L-selenome-thionine which showed a decrease in the number of DSBs after receiving the antioxidant compound (22); however, the effect of each antioxidant separately and also their efficiency have not been yet investigated in clinical trial conditions. In another study, Brand et al. investigated the effect of 9 antioxidants with different concentrations and at different incubation times according to the RDI and PI. Their results showed that ß-carotene, selenium, vitamins E and C, NAC, and Q 10 reduce the number of foci in vitro with 10 mGy dose (23). The findings of the current study showed that vitamin E can reduce the number of foci by 22% in comparison with the control group.

In two recent studies, it was reported that antioxidant compounds have more efficacy after 1 h incubation (22, 23); hence, in the current study, the effectiveness of vitamins E and C was examined 1 h after the administration.

There are few studies which have investigated the effects of antioxidants on radiation damages induced by a diagnostic dose. In a clinical trial, Stehli et al. reported that vitamin C and NAC led to a 66% decrease in the DSB number of the patients’ lymphocytes undergoing CT angiography who had received dose values more than 9 mSv. Never theless, these compounds had no significant effect on the DSB number of patients’ lymphocytes undergoing CT angiography with received doses less than 3 mSv (24). In the current study, the patients received the radiation dose values between 2.4 and 11.3 mSv, and the results showed that vitamin C had no significant effect on the number of DSB. In another study, Velauthapillai et al. investigated the effects of several antioxidants (ascorbate, N-acetylcysteine, lipoic acid, and beta-carotene) before technetium- 99m methylene diphosphonate (99mTc MDP) bone scans for cancer staging. In that prospective controlled trial study, antioxidant treatment was given to 5 consecutive patients before radiation exposure and these patients were compared with 5 patients without antioxidant treatment. Then, DNA damage was assessed in peripheral blood mononuclear cells before and after 99mTc MDP bone scans using fluorescently labeled γ-H2AX protein. Their findings indicated that there was a significantly higher number of gamma-H2AX foci/cell after radiation exposure in the control group in comparison with the antioxidant group. In addition, they reported that there was no statistically significant difference in number of gamma-H2AX foci/cell before or after radiation exposure in the antioxidant group, while the number of gamma-H2AX foci/cell was significantly higher in the control group (39). The differences between the current study and the study conducted by Velauthapillai et al. (25) lie in the different sources of exposure to the patients (abdomen/pelvic enhanced CT Scan vs. 99mTc MDP bone scan), number of participants, antioxidant type, and its concentration; hence, their results cannot be compared in details with the results of the present study.

There are some limitations in the present study which should be noted; first, the number of samples was small and further research is needed with larger sample sizes. Then, the combined effect of vitamins E and C was not examined in this study; however, Brand et al. reported that there was no additional effect resulting from the combination of several antioxidants (23). Although vitamin E reduced the number of DSBs in patients’ lymphocytes, its effect on solid tissues and on the prevention of cancer is still unknown.

Considering the importance of diagnostic X-ray dose values in increasing the population's cumulative dose, there is a serious necessity to reduce their complications in the long-term; hence, the use of antioxidants is suggested for this purpose. However, there is no complete clinical information to support this issue, because there are different antioxidants to study and their effects should also be investigated in different diagnostic modalities such as CT scan, angiography, and nuclear medicine.

## References

[1] United Nations Scientific Committee on the Effects of Atomic Radiation (2000). Sources and effects of ionizing radiation, UNSCEAR 2000 Report to the General Assembly with Scientific Annexes.

[2] Brenner DJ, Hall EJ (2007). Computed tomography--an increasing source of radiation exposure. N Engl J Med.

[3] Berrington de Gonzalez A, Darby S (2004). Risk of cancer from diagnostic X-rays: estimates for the UK and 14 other countries. Lancet.

[4] Aliasgharzadeh A, Mihandoost E, Masoumbeigi M (2015). Measurement of Entrance Skin Dose and Calculation of Effective Dose for Common Diagnostic X-Ray Examinations in Kashan, Iran. Glob J Health Sci.

[5] Brenner DJ (2012). Minimising medically unwarranted computed tomography scans. Ann ICRP.

[6] Task Group on Control of Radiation Dose in Computed T (2000). Managing patient dose in computed tomography A report of the International Commission on Radiological Protection. Ann ICRP.

[7] Hall EJ, Brenner DJ (2008). Cancer risks from diagnostic radiology. Br J Radiol.

[8] Mettler FA Jr, Bhargavan M, Faulkner K (2009). Radiologic and nuclear medicine studies in the United States and worldwide: frequency, radiation dose, and comparison with other radiation sources--1950-2007. Radiology.

[9] Jeggo PA, Lobrich M (2007). DNA double-strand breaks: their cellular and clinical impact?. Oncogene.

[10] Lobrich M, Rief N, Kuhne M (2005). In vivo formation and repair of DNA double-strand breaks after computed tomography examinations. Proc Natl Acad Sci U S A.

[11] Rothkamm K, Lobrich M (2003). Evidence for a lack of DNA double-strand break repair in human cells exposed to very low x-ray doses. Proc Natl Acad Sci U S A.

[12] Kuefner MA, Grudzenski S, Hamann J (2010). Effect of CT scan protocols on x-ray-induced DNA double-strand breaks in blood lymphocytes of patients undergoing coronary CT angiography. Eur Radiol.

[13] Andreucci M, Faga T, Pisani A (2014). Acute kidney injury by radiographic contrast media: pathogenesis and prevention. Biomed Res Int.

[14] Thomsen HS, Morcos SK (2003). Contrast media and the kidney: European Society of Urogenital Radiology (ESUR) guidelines. Br J Radiol.

[15] Grudzenski S, Kuefner MA, Heckmann MB (2009). Contrast medium-enhanced radiation damage caused by CT examinations. Radiology.

[16] Pathe C, Eble K, Schmitz-Beuting D (2011). The presence of iodinated contrast agents amplifies DNA radiation damage in computed tomography. Contrast Media Mol Imaging.

[17] Piechowiak EI, Peter JF, Kleb B (2015). Intravenous Iodinated Contrast Agents Amplify DNA Radiation Damage at CT. Radiology.

[18] Deinzer CK, Danova D, Kleb B (2014). Influence of different iodinated contrast media on the induction of DNA double-strand breaks after in vitro X-ray irradiation. Contrast Media Mol Imaging.

[19] Weiss JF, Landauer MR (2003). Protection against ionizing radiation by antioxidant nutrients and phytochemicals. Toxicology.

[20] Shirazi A, Mihandoost E, Mahdavi SR (2012). Radio-protective role of antioxidant agents. Oncol Rev.

[21] Mihandoost E, Shirazi A, Mahdavi SR (2014). Can melatonin help us in radiation oncology treatments?. Biomed Res Int.

[22] Kuefner MA, Brand M, Ehrlich J (2012). Effect of antioxidants on X-ray-induced gamma-H2AX foci in human blood lymphocytes: preliminary observations. Radiology.

[23] Brand M, Sommer M, Ellmann S (2015). Influence of Different Antioxidants on X-Ray Induced DNA Double-Strand Breaks (DSBs) Using gamma-H2AX Immunofluorescence Microscopy in a Preliminary Study. PLoS One.

[24] Stehli J, Fuchs TA, Ghadri JR (2014). Antioxidants prevent DNA double-strand breaks from X-ray-based cardiac examinations: a randomized, double-blinded, placebo-controlled trial. J Am Coll Cardiol.

[25] Safaei M, Jafarpour SM, Mohseni M (2018). Vitamins E and C Prevent DNA Double-strand Breaks in Peripheral Lymphocytes Exposed to Radiations from Iodine-131. Indian J Nucl Med.

[26] (2018). Erratum: The Radioprotective Effects of Curcumin and Trehalose Against Genetic Damage Caused By I-131. Indian J Nucl Med.

[27] Aliasgharzadeh A, Mihandoost E, Mohseni M (2018). A survey of computed tomography dose index and dose length product level in usual computed tomography protocol. J Cancer Res Ther.

[28] Huang X, Okafuji M, Traganos F (2004). Assessment of histone H2AX phosphorylation induced by DNA topoisomerase I and II inhibitors topotecan and mitoxantrone and by the DNA cross-linking agent cisplatin. Cytometry A.

[29] Rothkamm K, Balroop S, Shekhdar J (2007). Leukocyte DNA damage after multi-detector row CT: a quantitative biomarker of low-level radiation exposure. Radiology.

[30] Geisel D, Heverhagen JT, Kalinowski M (2008). DNA double-strand breaks after percutaneous transluminal angioplasty. Radiology.

[31] Kuefner MA, Hinkmann FM, Alibek S (2010). Reduction of X-Ray Induced DNA Double-Strand Breaks in Blood Lymphocytes During Coronary CT Angiography Using High-Pitch Spiral Data Acquisition With Prospective ECG-Triggering. Invest Radiol.

[32] Solomon R (2007). Contrast-induced nephropathy: update with special emphasis on patients with diabetes. Curr Diab Rep.

[33] Hill MA (2004). The variation in biological effectiveness of X-rays and gamma rays with energy. Radiat Prot Dosimetry.

[34] Buchegger F, Perillo-Adamer F, Dupertuis YM (2006). Auger radiation targeted into DNA: a therapy perspective. Eur J Nucl Med Mol Imaging.

[35] Nikjoo H, Emfietzoglou D, Charlton DE (2008). The Auger effect in physical and biological research. Int J Radiat Biol.

[36] Geisel D, Zimmermann E, Rief M (2012). DNA double-strand breaks as potential indicators for the biological effects of ionising radiation exposure from cardiac CT and conventional coronary angiography: a randomised, controlled study. Eur Radiol.

[37] Cheki M, Mihandoost E, Shirazi A (2016). Prophylactic role of some plants and phytochemicals against radio-genotoxicity in human lymphocytes. J Cancer Res Ther.

[38] Mihandoost E, Shirazi A, Mahdavi SR (2014). Consequences of lethal-whole-body gamma radiation and possible ameliorative role of melatonin. ScientificWorldJournal.

[39] Velauthapillai N, Barfett J, Jaffer H (2017). Antioxidants Taken Orally prior to Diagnostic Radiation Exposure Can Prevent DNA Injury. J Vasc Interv Radiol.

